# Intermediate care in caring for dementia, the point of view of general practitioners: A key informant survey across Europe

**DOI:** 10.3389/fmed.2022.1016462

**Published:** 2022-10-19

**Authors:** Clarisse Dibao-Dina, Caroline Oger, Tony Foley, Péter Torzsa, Vanja Lazic, Sanda Kreitmayer Peštiae, Limor Adler, Ana Kareli, Christian Mallen, Cindy Heaster, Gindrovel Dumitra, Donata Kurpas, Rita Viegas, Stéphanie Giezendanner, Victoria Tkachenko, Jan De Lepeleire, Rosario Falanga, Aristea Missiou, Aisling Jennings, Ferdinando Petrazzuoli

**Affiliations:** ^1^Department of General Practice, University of Tours, Tours, France; ^2^Department of General Practice, University College Cork, Cork, Ireland; ^3^Department of Family Medicine, Faculty of Medicine, Semmelweis University, Budapest, Hungary; ^4^Dom Zdravlja Zagreb-Centar, Zagreb, Croatia; ^5^JZNU Dom Zdravlja “Dr. Mustafa Šehoviæ”, Department of General/Family Medicine, Tuzla, Bosnia and Herzegovina; ^6^Sackler Faculty of Medicine, Tel Aviv University, Tel Aviv, Israel; ^7^Georgian Family Medicine Association, Tbilisi State Medical University, Tbilisi, Georgia; ^8^Primary, Community and Social Care, Keele University, Keele, United Kingdom; ^9^Department of Family Medicine, Faculty of Medicine, Riga Stradiņš University, Riga, Latvia; ^10^Romanian National Society of Family Medicine, Bucharest, Romania; ^11^Department of Family Medicine, University of Medicine and Pharmacy, Craiova, Romania; ^12^Department of Family Medicine, Wrocław Medical University, Wrocław, Poland; ^13^Department of Family Medicine, NOVA Medical School, Lisbon, Portugal; ^14^Centre for Primary Health Care, University of Basel, Basel, Switzerland; ^15^Department of Family Medicine, Shupyk National Healthcare University of Ukraine, Kyiv, Ukraine; ^16^Department of Public Health and Primary Care, General Practice, University of Leuven, Leuven, Belgium; ^17^Department of Primary Care, Azienda Sanitaria Friuli Occidentale, Pordenone, Italy; ^18^Research Unit for General Medicine and Primary Health Care, Faculty of Medicine, School of Health Sciences, University of Ioannina, Ioannina, Greece; ^19^Department of Clinical Sciences, Center for Primary Health Care Research, Lund University, Malmö, Sweden

**Keywords:** intermediate care, primary care, dementia, care giver, caregiver support

## Abstract

**Background:**

Intermediate care is often defined as healthcare occurring somewhere between traditional primary (community) and secondary (hospital) care settings. High quality intermediate care is important in dementia, may prevent caregiver burnout and also lead to optimal care for people with dementia. However, very little is known about the point of intermediate care for persons with dementia in Europe.

**Research questions:**

What intermediate care services exist and how are they utilized in the care of people with dementia in Europe?

**Objective:**

This study aims at describing the point of view of General Practitioners on intermediate care services for people with dementia across Europe.

**Methods:**

Key informant survey was sent to GPs via a self-developed questionnaire with space for open ended comments. 16 European countries participated to this cross-sectional mixed method study. Given the volunteer nature of the study, no minimum sample size requirements were applied to participation. Convenience sampling technique was used to address variations due to regional variations and regulations within the same country. Descriptive analyses of all intermediate care facilities groups by countries were performed. Qualitative analyses approach was used for the optional-free text to exemplify and/or complete the reasons contained in the closed response categories.

**Results:**

The questionnaire was sent to 16 European countries. 583 questionnaires were analyzed. The responding physicians were 48 (± 11) years old on average and they had been in practice for an average of 18 (+ /11) years. The types of intermediate care considered were integrated at-home services, respite and relief services, day care centers and nursing homes. Their availability was considered very inhomogeneous by the majority of respondents. The main benefits of intermediate care cited were better medical care for the patient (78%), better quality of life for the caregiver (67%), prevention of the caregiver burden (73%) and a break for the caregiver (59%). The reported difficulties were: accessing these facilities due to limited financial support (76%) and cumbersome administrative procedures (67%). Many other facets of our findings were captured in the qualitative themes that emerged.

**Conclusion:**

Intermediate care in Europe is diverse and heterogeneous. Major concerns of GPs are about the cost issues and the cumbersome administrative procedures to access them.

## Introduction

For many years policymakers have encouraged citizens to age at home. While many older adults live well independently, others with multi-morbidity and frailty rely on the support from family members, leading to a significant impact on the support-givers. The term “caregiver’s burden” is often employed to describe these negative consequences. The impact of caregiver burden includes neglected personal health, depression, anxiety, financial problems and employment losses (1–3). Caregivers of people with dementia have a higher risk of care-giver burnout and so are in particular need of support (4). These caregivers and their close relatives are also more vulnerable to social isolation and psychological distress resulting from the heavy demands of care-giving and the challenges of managing dementia, in particular the challenges of managing behavioral and psychological symptoms of dementia (4).

Serious concerns have been raised about a future shortage of family caregivers. Women’s increased labor force participation, the aging demographic, smaller families that are more geographically dispersed and the longer duration and increasing complexity of unpaid care-giving work are all factors that contribute to this shortage (5). In order to provide rehabilitation services, prevent prolonged hospital stays, and reduce readmission to hospitals among the elderly, aging societies have established intermediate care facilities, which involve both health and social care services in a variety of settings: rehabilitation units in hospitals nursing-led care inpatient units, post-acute care units in community hospitals, nursing homes, and patients’ own homes. Home care encompasses a broad range of services including needs assessment, personal care provision, leisure activities and rehabilitation at home (6, 7).

Intermediate care is an increasingly popular concept in health care, which may offer attractive alternatives to hospital care for elderly patients. A prerequisite for research is the agreement on the definition of a concept; however, there is no accepted definition of the term “intermediate care.” Intermediate care conveys little meaning other than being about care that is “in between.” Several very different definitions of intermediate care are in use. Broadly speaking, intermediate care is often defined as healthcare occurring somewhere between traditional primary (community) and secondary (hospital) care settings (8); according to other authors (9) intermediate care refers to “services or activities concerned with patients’ transition between hospital and home, and from medical social/dependence to functional independence.” Intermediate care provides a bridging function between hospital and home, and is geared “toward promoting faster recovery from illness, preventing unnecessary acute hospital admissions, supporting timely hospital discharge and, most of all, enabling people to retain their independence for as long as possible” (10).

In UK the criteria of Intermediate Care were established by the Department of Health in 2001 (11). Intermediate care can vary from in-home services to well-equipped nursing homes. High quality intermediate care is important in the care of dementia and may prevent caregiver burnout. Intermediate care is delivered by those health services that do not require the resources of a general hospital but are beyond the scope of the traditional primary care team. In PubMed “intermediate care facilities” are institutions that provide health related care and services to individuals who do not require the degree of care which hospitals or skilled nursing facilities provide, but require care and services above the level of room and board (12). It is crucial to have a clear definition of this term in order to have a proper development of appropriate services that will meet the needs of patients and family carers, for healthcare services to plan, for governments to spend wisely.

## Key concept

### Different types of intermediate care

#### Integrated at-home services

**FIGURE 1:**
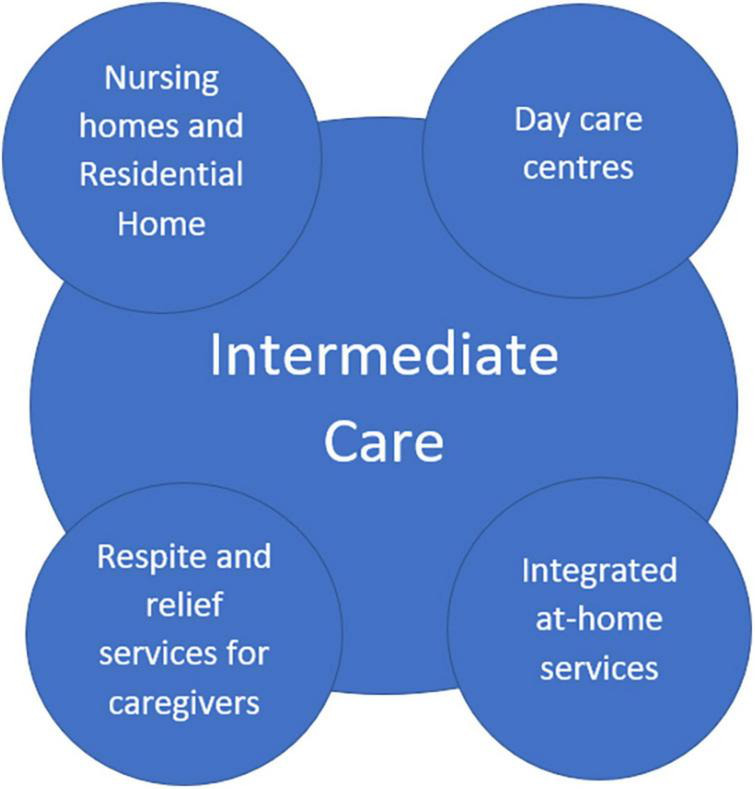
The different types of intermediate care.

The patient is cared for at home just for a few hours by social workers people from the voluntary sector et cetera, and visited at home by doctors, nurses. The familial/informal caregiver is supported in terms of clinical tasks to do but still has a 24 h/day commitment and he/she does not have free time. The caregiver has to give up many things in his/her life (Figure 1).

Integrated care is described as “the management and delivery of health services so that clients receive a continuum of preventive and curative services, according to their needs over time and across different levels of the health system” (13).

Integrated care is associated with greater client satisfaction, increased use of community based services, and reduced hospital days, however, the clinical impacts on persons with dementia and their carers are not known (14).

#### Respite and relief services for caregivers

Respite care is short-term relief for the caregiver. The patient is cared for at home for part of the day by social workers people from the voluntary sector paid carers et cetera, and visited at home by doctors, nurses. The familial/informal caregiver is supported not only in terms of clinical tasks to do but he/she is given also free time to get out of the house because the task of caregiver is taken over temporarily by someone else. Respite care offers caregivers a temporary break from their daily routine and the stress from caregiving (15). Utilization of respite care has been found to be generally low in different countries despite high levels of need (16).

#### Day care centers

The patient is taken to a different place but just for a limited period of time during the day. During this time the familial/informal caregiver is free to do what he/she wants.

Day care is assumed to promote independence in home-based people with dementia, increase wellbeing, and improve social stimulation. Between 10 and 18% of people with dementia in the community are utilizing the service internationally (17). There is significant variation between countries and across the same country in the existing capacity of day care centers to cater for people with dementia (18).

Day care for older people can be defined in several ways; a “social model” in which the centers aim to provide socialization and activity and a “medical model” in which health and rehabilitation services are provided (19).

Some authors have showed that day care can have a positive influence on patients’ physical functioning, cognition, wellbeing and situation at home because they were provided with social stimulation, meals and activities. Day care helped maintain a rhythm and structure in daily life (20).

#### Nursing homes and residential home

(In the nursing home the staff is down the hall 24/7. In the residential home the staff is involved only for a limited period of time a day). The patient is staying at a different location all the time. No need for familial/informal caregiver. Informal caregivers often describe aspects of care that led to both positive and negative experiences with and perceptions of nursing home care (21).

## Aim

This study aims to identify the different modalities of intermediate care and how it is provided in Europe and to describe GPs’ views on the advantages and disadvantages of different intermediate care services for people with dementia in Europe.

## Materials and methods

### Study design and setting

This cross-sectional study is based on a key informant survey from 16 member countries of the European General Practice Research Network (EGPRN). EGPRN operates under the umbrella of WONCA (World Organization of National Colleges, Academies and Academic Associations of General Practitioners/Family Physicians).

### Procedure

The study started off as an idea of the EGPRN Education Committee researchers and primary care professors from the University of Tours, France, University College Cork, Ireland and the University of Louvain, Belgium. The steering committee (CDD, FP, AJ, JDL) of this project, called “Intermediate care for dementia in Europe,” developed a semi-structured questionnaire with 20 questions. It contained quantitative/qualitative questions, some in Likert scale-type and open-ended questions as well.

### Development of the questionnaire

The steering committee of this project, called “Intermediate care,” developed a semi structured questionnaire with 20 questions, 14 of these included free text comments.

The first draft of the questionnaire was based on the research objectives through an extensive literature review. Subsequently, a panel of PHC experts and methodology experts used a Delphi process to evaluate the validity of the items and the length of the questionnaire, formulate suggested changes, and identify missing items. The research team then discussed all feedback until consensus was reached, and a final version of the questionnaire was developed.

### Validity

The psychometric properties of the questionnaire were assessed both quantitatively and qualitatively, focusing on validity as a theoretical construct and as an empirical construct. Regarding validity as a theoretical construct, face validity and content validity were tested. During the development of the questionnaire, face validity, i.e., whether the questionnaire measures at first glance what it purports to measure, and content validity, i.e., whether the items adequately represent the entire domain that the questionnaire attempts to measure, were tested. In each case, this was done by EGPRN Primary health care experts, all international authorities in the field of health care. Construct validity, i.e., the extent to which the items in the instrument relate to a relevant theoretical construct, was improved by using the results in the first step of the development process. Given that full randomization was not possible in all countries, a sampling bias may exist, which might have affected external validity. Some strategies were implemented to minimize the potential biases encompassed by conducting multicenter surveys. Each partner undertook the translation (and back-translation) and cultural adaptation of the questionnaire first, and then after resolving terminology issues, the collaborators reached the harmonized version of the questionnaire, with consideration of local arrangements and definitions. This rigorous development of the questionnaire is a strength of the study.

### Participants

EGPRN National coordinators from 16 European countries, (Bosnia, Croatia, Georgia, Greece, France, Hungary, Ireland, Israel, Italy, Latvia, Poland, Portugal, Romania, Switzerland, United Kingdom, and Ukraine), agreed to coordinate the survey in their countries.

A convenience sampling technique was used whereby national coordinators (members of EGPRN) chose informants from different geographical regions within their own country. The informants were contacted directly by the national coordinators by email; the participants were required to be primary healthcare doctors. The informants were asked to give the general view of the attitude of P in their country. Since we used a convenient sample of informants, the representativeness of primary care personnel for each country may be questionable although we tried to achieve geographical distribution especially in big countries. The national coordinators tried to avoid bias and recruit practicing primary care physicians with different interests, and not necessarily in dementia care. This type of recruitment strategy has been currently used in many collaborative recent studies in Primary care.

### Sample size

Exact data on the population of general practices in every partner country was not available to calculate the target sample size, and, additionally, given the volunteer nature of the study, no minimum sample size requirements were applied to participation. For this reason, we did not focus on presenting individual country-level data in detail. Given the potential volunteer bias and the cross-sectional survey design, direct assessment of causal relationships was not possible.

### Main outcome measures

The questionnaire comprised 20 questions including sociodemographic variables and length of clinical experience (Supplementary material). The data were collected using the online survey tool Google Form.

The authors planned descriptive analysis of the quantitative data.

### Analysis

Six hundred and six doctors responded to the questionnaire. 16 responses were excluded because the respondent was not a general practitioner or had not answered the first question. On the other hand, 7 responses were excluded for duplicates. 583 responses were finally analyzed.

To describe baseline characteristics, proportions were calculated for dichotomized or categorized data, and means for continuous data. Median and interquartile range were utilized for the analysis of Likert scale data. Statistical analyses were performed in Microsoft Office Excel 2010 software.

### Qualitative analysis

Because responses were limited to short sentences for the open-ended questions, a brief conceptual qualitative content analysis was conducted. Responses (direct quotes) from GPs were independently reviewed by two members of the research team (22, 23).

## Results

Six hundred and six doctors responded to the questionnaire. Sixteen responses were excluded because the respondent was not a general practitioner or had not answered the first question. On the other hand, 10 responses were excluded for duplicates (3 in Israel). Five hundred and eighty three responses were finally analyzed. Response rate was above 50%.

### Characteristics of participants

#### Physician characteristics: 61% (357) of the participants were women and 39% (226) men

The average age is 48 years with a standard deviation of 11 years.

All the characteristics of the surveyed population can be seen in Table 1.

**TABLE 1:** Characteristics of participants.

	Number of participants	Men	Women N (%)	Age Med (Q1-Q3)^a^	Urban	Semi-rural	Rural	Seniority Med (Q1-Q3)
Bosnia	29	4 (14%)	25 (86%)	50 (36–54)	17 (59%)	6 (21%)	6 (21%)	17 (6–25)
Croatia	19	3 (16%)	16 (84%)	31 (30–39.5)	13 (68%)	6 (32%)	0	6 (4.5–11)
France	50	28 (56%)	22 (44%)	50 (39–59)	13 (26%)	27 (54%)	10 (20%)	16.5 (8.25–29.75)
Georgia	22	0	22 (100%)	41 (38. 25–51.25)	5 (23%)	4 (18%)	13 (59%)	11.5 (7.5–20)
Greece	23	8 (35%)	15 (65%)	43 (38.5–45)	8 (35%)	7 (30%)	8 (35%)	5 (5–9.5)
Hungary	24	14 (58%)	10 (42%)	47.5 (40–53.5)	22 (92)	2 (8%)	0	16 (11.75–22.25)
Ireland	34	20 (59%)	14 (41%)	49 (45–59)	16 (47%)	12 (35%)	6 (18%)	20 (16–26)
Israel	28	11 (39%)	17 (61%)	46.5 (40.3–55)	23 (82%)	1 (4%)	4 (14%)	14.5 (5.75–23.5)
Italy	31	25 (81%)	6 (19%)	61 (61–66)	11 (35.5%)	9 (29%)	11 (35.5%)	35 (26–37.5%)
Latvia	20	2 (10%)	18 (90%)	49 (44–60)	13 (65%)	3 (15%)	4 (20%)	20 (7.3–23)
Poland	37	19 (51%)	18 (49%	47 (32–53)	22 (60%)	9 (24%)	6 (16%)	18.5 (5.5–21.3)
Portugal	40	13 (33%)	27 (68%)	43 (37.8–57.3)	33 (82.5%)	5 (12.5%)	2 (5%)	11 (9.5–31)
Romania	105	8 (8%)	97 (92%)	54 (48–60)	66 (63%)	6 (6%)	33 (31%)	21 (17–28)
Switzerland	53	42 (79%)	11 (21%)	55 (47–59)	31 (58%)	16 (30%)	6 (11%)	18 (10–22)
United Kingdom	34	17 (50%)	17 (50%)	42 (36.3–47.5)	22 (65%)	9 (26%)	2 (6%)	9 (5–13.8)
Ukraine	34	12 (35%)	22 (65%)	38 (32–44.8)	20 (59%)	9 (26%)	5 (15%)	11.5 (7–18.8)
Total	**583**	**226 (39%)**	**357 (61%)**	**49** (39–58)	**335 (57%)**	**131 (22%)**	**116 (21%)**	**17** (8–25)

^a^Median (1st quartile–3rd quartile).

#### Setting and seniority of work

The doctors surveyed mainly practiced in urban areas 57% (335) of the participants. The semi-rural and rural practices consisted of 22% (131) and 20% (116) of doctors, respectively. One participant did not answer this question.

At the time of the questionnaire, physicians had practiced medicine on average for 18 years with a median of 17 years.

### What do you think might be the main benefits of intermediate care for a patient with dementia?

Respondents identified the main advantages of intermediate care as better medical care for the patient (78%), better quality of life for the caregiver (67%), prevention of caregiver burnout (73%) and a break and assistance for the caregiver (59%).

**FIGURE 2:**
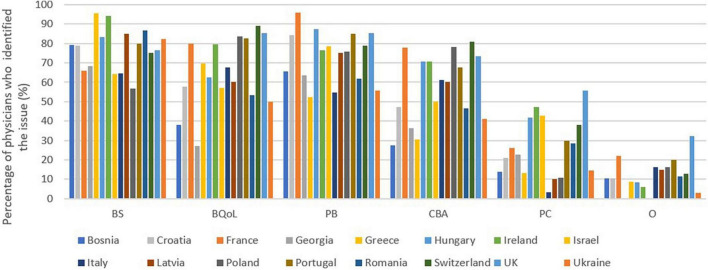
Intermediate care: Main benefits declared by general practitioners. BS, Better support; BQoL, Better quality of life; PB, Prevention of burnout; CBA, A break and assistance for the caregiver; PC, Respect for the patient’s choice; O, Others.

Respect for the patient’s choice was identified by 27% of physicians, but in a heterogeneous manner depending on the country. The presence of other benefits was reported in 14% of cases. The full picture of the responses by country is available in Figure 2.

**Qualitative analysis: Analysis of qualitative data revealed three main themes:**

1. **Improved care:** *“It would certainly improve the current care of such patients, as well as their caregiver*… *and would also have a positive impact on their interpersonal relationships”* Croatia.
2. **Enhanced quality of life:** *“Better quality of life for both the patient and the caregiver and the rest of the family”* Bosnia-Herzegovina.
3. **Reduced costs to the healthcare system:** *“It is cheaper than hospital care. It avoids bouncing in and out of inappropriate hospital”* UK.

### What do you think are the main disadvantages of intermediate care in a patient with dementia?

According to the doctors, the main disadvantage seems to be the high cost for the family (66%). The second disadvantage (51%) is the “disorientation and exacerbation of behavioral and psychological symptoms of dementia because the patient was transferred to another living environment.” The third main drawback identified is the feeling of shame with 46% of doctors having reported it.

The cost to society is a disadvantage for 36% of physicians.

Poor medical care, decline in the quality of life of the caregiver and poor prognosis of the patient’s state of health do not seem to be concerns for the doctors because less than 15% checked these answers.

**FIGURE 3:**
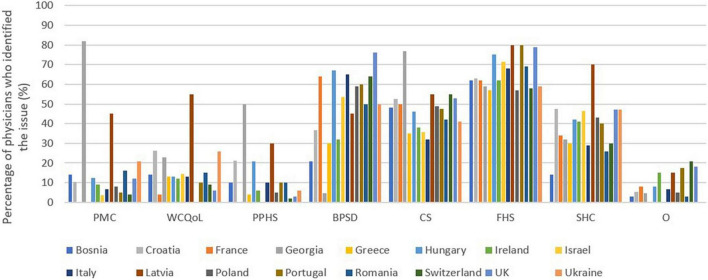
Intermediate care: Main disadvantages declared by general practitioners. PMC, Poor medical care of the patient; WCQoL, Decrease in the quality of life of the caregiver; PPHS, Poor prognosis of the patient’s state of health; BPSD: Disorientation and exacerbation of behavioral and psychological symptoms of dementia because the patient has been transferred to another living environment. CS: Feeling of shame on the part of the caregiver (s) for not being able to take care of the patient (s) themselves (“natural” obligation according to social conventions). FHC, High cost for the family; SHC, High cost for society and the healthcare system; O: Others.

The breakdown of responses by country is available in Figure 3.

**Qualitative part: analysis of qualitative data revealed two main themes:**

1. **Funding:** *“There is no doubt that the issue of funding and fundability is a disadvantage*,” Hungary and *“Long-term care is a considerable financial burden for relatives, which only stops when the assets are used up and the general public has to assume the uncovered costs*,” Switzerland.
2. **High staff turn-over:** *“Turnover of staff causing lack of rapport and familiarity,”* Ireland and *“High staff turnover and subsequent issues with continuity of care,”* UK.

### What kind of intermediate care is available in your country?

**FIGURE 4:**
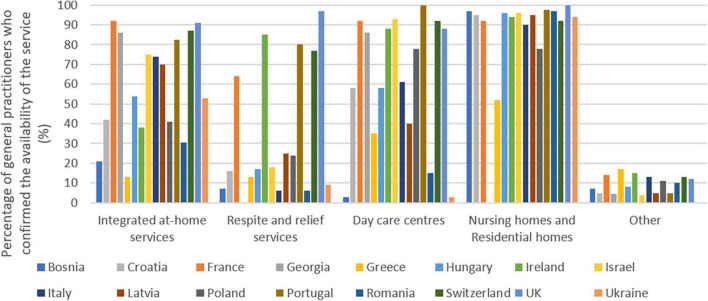
Availability of intermediate care services.

The results are summarized in Figure 4.

There are big differences among countries in the availability of the different form of intermediate care a part the nursing homes. For the respite and relief services in Bosnia (7%), Croatia (16%), Greece (13%), Hungary (17%), Italy (6%), Israel (18%), Latvia (25%), Poland (24%), Romania (6%), Ukraine (9%) less than 50% of the respondents declare the existence of such kind of services.

Regarding the integrated care services countries below 50% were Bosnia (21%), Croatia (42%), Greece (13%), Ireland (38%), Poland (41%), Romania (30.5%). Regarding the day-care centers, countries with a positive response below 50% were Bosnia (3%), Greece (35%), Latvia (40%), Romania (15%), Ukraine (3%).

Regarding the nursing homes countries with a positive response below 50% was only Georgia (0.0%).

**Qualitative part: Other forms of intermediate care structures, included:**

1. **Foster families:** Croatia and France.
2. **Voluntary organizations:** *“Support from charities and community groups,”* UK and *“Volunteers present at home for a few hours/day*,” Switzerland.

### Are intermediate care services homogeneously available in your country?/(homogeneously means: no difference between regions and no difference between rural and urban setting)

**FIGURE 5:**
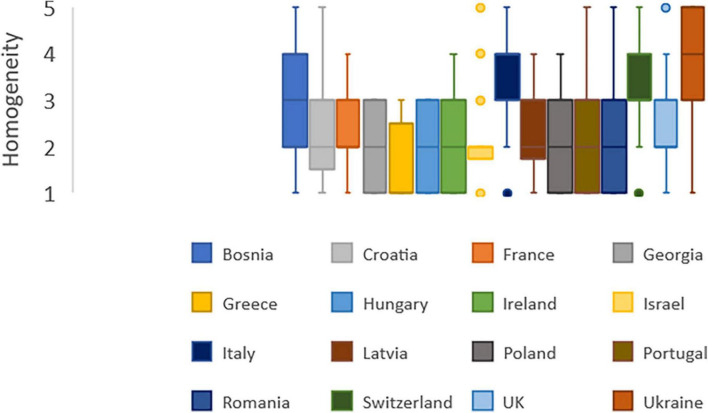
Intermediate care services: Homogeneity of diffusion. 1: not at all homogeneous. 5: completely homogeneous.

The median of responses is 1 for Greece, the majority of Greek respondents (12 out of 23 responses) find the availability of intermediate care very heterogeneous in their country (Figure 5).

The median of responses is 4 for Italy and Ukraine, indicating intermediate care available in a rather homogeneous manner.

Four countries stand out for their better homogeneity: Bosnia Herzegovina, Italy, Switzerland and Ukraine. Indeed, Bosnia Herzegovina and Italy have a median at 3 with a 3rd quartile at 4.

For the majority of countries, the median is 2. This is the case for France, Georgia, Hungary, Ireland, Latvia, Poland, Portugal, Romania, and UK.

**Qualitative part: From analysis of qualitative data two themes emerged:**

1. **A rural-urban divide:** *“The rural environment is disadvantaged in all respects*,” Romania and *“More often, access in cities is better*,” Poland and *“In rural areas, social services are less accessible*,” Latvia and *“In urban areas, it is more accessible*,” Bosnia and Herzegovnia and *“In rural locations, mainly in mountain villages, there are not at all intermediate care services*,” Greece.
2. **The social deprivation effect:** *“Wealthy councils have better care, there are long waiting lists for government care*,” Latvia and *“Gaps most commonly in areas of deprivation but even that is not consistent,”* Ireland.

### Are these two types of intermediate care integrated at-home services/respite and relief services for caregivers, described in full in question provided for free in your country? (Free means that the patient is not charged, or that he/she is reimbursed by the health care system)

Two answers were uninterpretable because they were not formulated according to the available scale.

For this question and the following one, the scale went from 1 (not at all) to 3 (completely free or fully reimbursed).

Free home care and respite facilities were reported on average in only 9% of cases, with a minimum in Latvia of 0% and a maximum in Georgia with 27.3% of responses.

In France, Georgia, Ireland, Israel, Italy, Latvia, Poland, Portugal, Switzerland and the United Kingdom more than 50% of doctors described partial reimbursement for home care and respite facilities.

**FIGURE 6:**
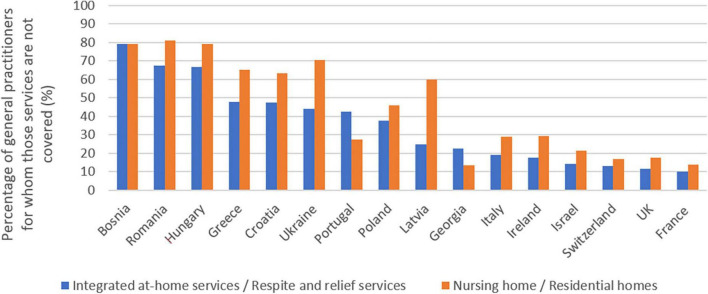
Intermediate care services: subsidization by the healthcare system.

The responses concerning the total lack of support for home care and respite facilities are summarized in Figure 6.

### Do families receive economic support when a patient is admitted to a nursing home or long-term care facility (type 3 and 4 in question 1) in your country?

Like the precedent question, two answers were uninterpretable because they were not formulated according to the available scale. The scale used was identical to the one mentioned in previous question.

Full coverage of retirement homes and assisted living facilities costs seems even rarer than for home care and respite facilities: only 5.3% of respondents checked this answer. In Georgia however, this response was checked in 45.4% of cases out of a sample of 22 doctors. In other countries, it was checked between 0 and 11% of cases.

Note that no doctor from Georgia has reported the availability of nursing homes or residences in their country to the question concerned.

Only France, Ireland, Israel, Italy, Portugal, Switzerland and the United Kingdom also reported partial reimbursement in more than 50% of cases for nursing homes and assisted living facilities.

The responses concerning the total lack of support for assisted living facilities are also summarized in Figure 6.

Nursing homes and serviced residences seem to be less well supported than home care and respite facilities, with the exception of Georgia and Portugal.

### Does your country have written standards or guidelines for admission to any form of intermediate care?

49.7% of doctors responded that there are no written standards or guidelines for access to any type of intermediate care in their country.

Countries where there appear to be no standards according to doctors are Bosnia Herzegovina, Croatia, France, Georgia, Greece, Hungary, Ireland, Israel, Latvia, Romania, Switzerland, and Ukraine.

36.7% of respondents believe that there are written standards or guidelines in their country. 13.6% checked “other.”

However, in Italy, Poland, Portugal and the UK, more physicians confirmed the existence of standards or guidelines.

**Qualitative part: Three themes emerged from analysis of the qualitative data:**

1. **GP’s lack awareness of admission guidelines**: *“I honestly have not come across anything like that,”* Hungary and *“I don’t know*,” Portugal and *“I am not aware of any guidelines on this,”* Switzerland.

**2. Multi-disciplinary assessment of clinical need: “***Multidisciplinary evaluation units that assign a score, relative to the patient’s need,”* Italy and “*an examination by a psychiatrist, psychologist and internal medicine specialist is required for admission to the institution,”* Bosnia and Herzegovnia and *“The family doctor and the manager of the home should score the patient’s current condition and the level of social care,”* Hungary and *“Assessed by the primary care doctor and the home/service staff on the basis of self-care grades,”* Hungary and *“Must be assessed by age care assessment team with consultant geriatric doctor*,” Ireland and *“Admission is based on evaluation by the District Physician and subsequent introduction of the patient to the regional ranking list”* and *“GP report with the opinion of the Psychiatrist*,” Portugal.

1. **Funding: “***In the private sector the determinant is having money to pay the costs,”* Portugal and *“Personal connections are more important, or geographical availability and of course, money,”* Hungary.

### What is in your opinion the general attitude of family caregivers toward admitting their relative to a nursing home in your country?

The question concerned the feelings of caregivers regarding the admission of their loved one to a retirement home in the country of the surveyed. The scale ranged from 1 (happy) to 10 (ashamed).

**FIGURE 7:**
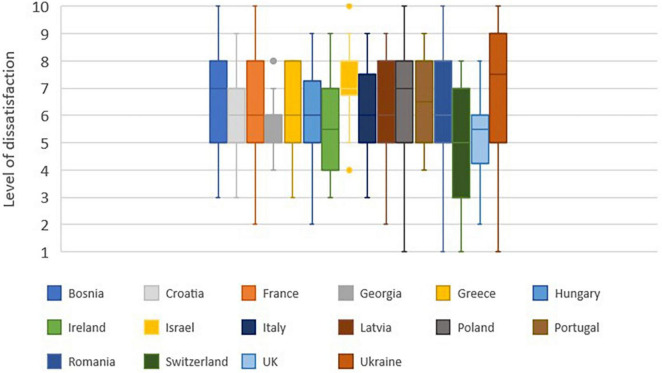
Level of caregiver dissatisfaction of intermediate care. 1: happy. 10: ashamed.

The opinion of respondents is unanimous across the countries with a median systematically greater than or equal to 5 (Figure 7).

**Qualitative part: Five themes emerged following analysis of the qualitative data:**

1. **Shame:** *“experienced as a shame or failure*,” France, and *“Guilt, feelings of failure, fear*,” France and *“Some are ashamed but, again, this is perhaps not the most common reaction*,” Ireland and *“families are not proud of placing the charges in nursing homes, they hide it from their friends*,” Poland.
2. **Sadness:** *“Probably sadness/anguish for not being able to care,”* Portugal and *“It is a painful choice,”* Italy and *“Most feel sad but have reached the end of their coping strategies by this point*,” UK.
3. **Guilt:** *“The feeling of guilt is great*,” Poland and “Switzerland and *“It is considered that you are a bad son or daughter if you cannot take care of your parents on your own,”* Ukraine.
4. **Relief:** *“It is thought that their relative may have better supervision and treatment in a nursing home than at home*,” Greece and *“At a point of burnout, it is accepted,”* Hungary and *“Many families are relieved by the time if admission despite wishing things could be different*,” Ireland and *“Relief since they can no longer make it on their own*,” Italy and “…*appreciate the fact that they are relieved of the burden of taking care of the sick, which they are not obliged to share with such a center,” Poland and “It varies from family to family, but in most cases it is a relief*,” Portugal.
5. **Family-dependent heterogenous responses**: “…*it is different for each individual,”* Hungary and *“Varies hugely from family to family*,” Ireland and “…*very dependent on relatives*,” Latvia and *“family dependent and culture dependent*,” UK.

### Do you as a GP have the power to admit a patient to any form of institutional support of intermediate care?

Respondents from Bosnia and Herzegovina (72%), Croatia (63%), France (70%), Greece (61%), Ireland (73%), Israel (54%), Latvia (50%), Poland (57%), Romania (79%), and the United Kingdom (50%) consider that they do not have the power to admit a patient to any form of institutional support of intermediate care.

Responses from general practitioners in Georgia (73%), Hungary (71%), Italy (45%), Switzerland (85%), and Ukraine (59%) showed that GPs have this power. Note that in Italy, 20% of doctors ticked the answer “Other.”

Qualitative part: The themes that emerged from analysis of the qualitative data indicated that GPs do not have the power to admit a patient to any form of institutional support of intermediate care. Three themes emerged.

1. Cost

“It depends on the urgency of the placement and the cost to the patient’s family,” Italy

“It depends on financial means,” France.

1. Bed Availability

“Frequent refusals due to lack of available space,” France.

“Can suggest the need, but can’t ensure availability of beds,” Ireland.

1. Governance

“…does not depend on the doctor, because the admission commission is independent of the doctor,” France.

“I can suggest. Intermediate care is given by the social services, not medical,” Israel.

“I Need authorization from a specialist working in the public health system,” Italy.

### Do you, as a GP, need an approval from a secondary care specialist or any other authority to admit a patient to any form of institutional support?

In some countries non-medical administrative staff such as health service economists need to approve the admission of the patient to these forms of institutional support.

In Croatia (74%), France (86%), Hungary (75%), Poland (62%), Portugal (60%), Switzerland (92%) and the United Kingdom (59%), respondents denied the need of the approval of a secondary care specialist or other authority in order for a patient to receive intermediate care.

Conversely, in Bosnia Herzegovina (62%), Georgia (82%), Greece (48%), Ireland (68%), Israel (69%), Italy (71%), Latvia (50%), Romania (67%), and Ukraine (82%), respondents stated that GPs do need the approval of a secondary care specialist or some other authority.

Qualitative part: There was much variability with Countries.

“Complicated procedure,” Bosnia and Herzegovina

“Once again, it depends, for some home care or some,” France

“In some cases, they seek psychiatric advice, but in most cases they do not,” Hungary

“… in some situations Yes; in others No,” Ireland.

### How would you rate the quality of intermediate care for people with cognitive impairment in your country?

The scale ranged from 1 (low quality) to 5 (high quality).

The average across all countries is 2.48 with a standard deviation of 1.04.

The outcome for this question is available in Table 2.

**TABLE 2:** Assessment of the quality of care by general practitioners by country.

Country	Median	Minimum	Maximum	1^er^ Quartile	3^e^ Quartile
Bosnia	**2**	1	3	1	2
Croatia	**3**	1	5	2	3
France	**3**	1	4	2.25	4
Georgia	**2**	1	4	2	3
Greece	**2**	1	3	1	2
Hungary	**2**	1	4	1.75	3
Ireland	**3**	1	5	2	3
Israel	**3**	1	5	2	3.3
Italy	**3**	1	4	2	4
Latvia	**2**	1	3	2	2
Poland	**2**	1	4	1	3
Portugal	**2.5**	1	3	2	3
Romania	**2**	1	4	1	2
Switzerland	**4**	2	5	4	4
UK	**3**	2	4	2.25	3
Ukraine	**3**	1	4	2	3

### What are in your opinion the main issues related to intermediate care for persons living with dementia in your country or in your area?

The major issues reported are limited financial support (76%) and access difficulties with restrictive administrative procedures (67%).

Equally important issues are the poor quality of service (43%), stigmatization for the family (41%). The least important issue is the feeling that the situation has worsened in recent years due to cuts in health spending (35%). 16% of physicians checked “other.”

**FIGURE 8:**
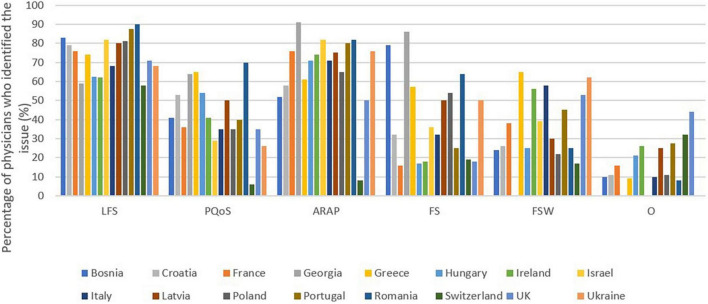
Main issues with intermediate care. LFS, Limited financial support; PQoS, Poor quality of service; ARAP, Access difficulties with restrictive administrative procedures; FS, A stigma for the family (for the retirement home); FSW, A feeling that the situation is worsening in recent years, due to cuts in health spending; O, Other.

The complete picture of the responses by country is available in Figure 8.

### Are general practitioners in your area educated and trained to familiarize themselves with and manage intermediate care services?

51% of doctors consider they are not at all trained and 44% moderately trained. Only 5% of doctors think they are very well trained.

Switzerland (13%) and Ukraine (26%) are the only two countries to exceed 7.5% of responses in favor of very good training.

Doctors from France (66%), Greece (57%), Hungary (75%), Israel (54%), Italy (61%), Portugal (52.5%), Romania (80%), and Ukraine (44%) consider that GPs are not trained at all.

For the other countries, the majority of doctors think they are moderately trained. This is the case of Bosnia Herzegovina (59%), Croatia (53%), Georgia (82%), Ireland (65%), Latvia (55%), Poland (54%), Switzerland (60%), and the United Kingdom (65%).

### In your area are GPs regularly updated on the intermediate care services available?

Two countries stand out with more than 20% of respondents who consider that general practitioners are regularly informed about the available intermediate care services: Switzerland (26%) and Ukraine (23%). In the other countries, less than 7.5% of physicians ticked this answer.

The countries where more than half of doctors think they are not at all informed are France (54%), Greece (70%), Hungary (75%), Israel (50%), Italy (61%), Latvia (55%), Poland (65%), Portugal (72.5%), Romania (90%).

Doctors from Bosnia and Herzegovina (52%), Croatia (58%), Georgia (86%), Switzerland (53%), United Kingdom (50%) mostly chose the intermediate level of response 2.

Half of the doctors in Ireland said they were not at all informed and the other half that they were moderately informed.

Responses in Ukraine show significant heterogeneity between physicians. Indeed, it is one of the countries where the rate of doctors who think they are well informed is the highest (23%). However, 41% of physicians responded that they were not at all informed compared to 35% for the intermediate response.

## Discussion

In the literature the effectiveness of strategies to reduce family care giver burden gives controversial results. A systematic review conducted by Schoenmakers et al. (24) demonstrated, in accordance with other qualitative reviews, the weak evidence that supporting family caregivers could be beneficial.

### Advantages and disadvantages of intermediate care

According to our respondents better medical care for the patient, better quality of life for the caregiver and prevention of caregiver burnout seems to be the main advantages.

the main disadvantages seem to be the high cost for the family (66%), the “disorientation and exacerbation of behavioral and psychological symptoms of dementia because the patient was transferred to another living environment.” In case of nursing home or day centers, and the feeling of shame of the family caregiver.

According to the literature attending a day care center provides the opportunity for social interaction and a sense of structure and routine (20, 25), and day care has been shown to provide people with dementia with a range of benefits. These include increased wellbeing (26, 27); better sleeping habits (27, 28); reduced neuropsychiatric symptoms and use of psychotropic drugs (28, 29); and, reduced family carer stress (30).

Respite services are also significantly associated with decreased levels of carer burden (29). Regarding the patient with dementia positive aspects described in the literature included health assessments received during respite, activities encouraging stimulation, socialization and keeping active, improvement in self-esteem, physical health, cognition and conversation, enjoyment of respite, the provision of a safe environment and the chance to have time out of the house and away from family care (31, 32).

For the family, the admission to a nursing home might also indicate an opportunity to receive end-of-life care in the facility (33).

Because a change in the place of care and caregivers adversely affects cognitive functioning (34, 35), intermediate care in residential care settings might cause problems in terms of development of Behavioral and psychological symptoms of dementia (BPSD). Moreover some elderly persons are admitted to geriatric intermediate care facilities because an appropriate facility is not available (36), and nursing homes sometimes reject admission for people with challenging behaviors (37).

Older age, higher level of care need, and several medical conditions (including dementia and dysphagia) are usually associated with lower likelihood of discharge to home, because these medical conditions can make it difficult to care for older adults at home (38).

People with dementia are often reluctant to go to a day care center, which poses a dilemma for care givers (39, 40). Centers that cater for people with dementia may also have restrictions on enrolment; incontinence and disruptive behavior are cited as the most common restrictions, and not surprisingly, therefore, day care utilization rates among people with dementia tend to be low (17).

### Availability of the different types of intermediate care

In our survey a part the nursing home there are big differences among countries in the availability of the different types of intermediate care.

Good quality community care should be accessible to all people living with dementia (41). Hospitalization or nursing home admission of people with dementia may reflect inequities in availability of community care (41). The types and availability of home care services differ by country. A review of American studies reporting service use estimated that 46.7% of community-dwelling people with dementia used in-home health aide services during a 1-year period and homemaker assistance was used by around 23–36.8% (17).

### Geographic variation

Apart from Greece, the majority of responses showed a rather homogenous availability of these services within the single countries respondents sometimes complained of poor availability in rural environments.

According to the literature, in Ireland day care centers, in many parts of the country, have limited capacity to provide a service for people with dementia who live in their catchment area (18). As the number of people with dementia increases, investment in day care centers should be targeted to areas where need is greatest. Despite the attempt, there is no universal access to care, the services available to each GP participant often vary according to geographic sites (18, 42). Interventions are needed to support families of people with dementia, because they incur the most dementia care related costs (43).

### Financial support to the family

Regarding the economic support to family for intermediate care, this issue is raised by most of our respondents. Free home care and respite facilities were reported on average in only 9% of cases, and the coverage of the costs of nursing homes and assisted living facilities seems even rarer.

According to the literature in a recent qualitative study conducted in Ireland, GPS pointed at the scarcity of funding as a barrier to patients and their carers in accessing secondary services; they also complain that community nurses are not enough and sheltered accommodations need to become widely distributed (44).

### Guidelines for admission to any form of intermediate care

**FIGURE 9:**
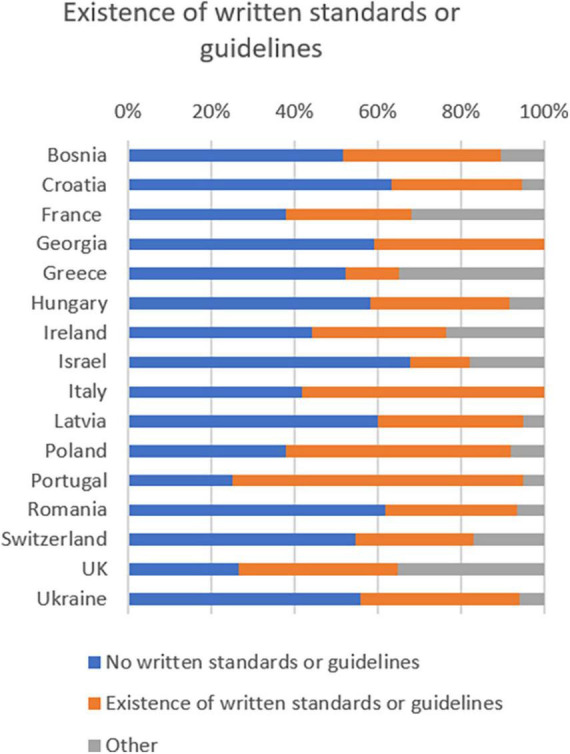
Intermediate care: existence or written standards or guidelines.

In our survey many respondents denied the existence of written guidelines (Figure 9).

### Attitude of family caregivers

According to our respondents, caregivers are not completely keen to admit their relatives to a nursing home.

Respite care is a cornerstone service for the home management of people with dementia and it is used by carers to mitigate the stress related to the demands of caring by allowing time for them to rest and do things for themselves, thus maintaining the caring relationship at home and perhaps forestalling long-term placement in a residential aged care facility (16). Unfortunately, its uptake by carers of people with dementia remains relatively low (16).

In a number of interview studies, both quantitative and qualitative, carers appeared conflicted about giving themselves permission to initially use respite services (45). Guilt from perceptions of abandoning the person with dementia, failure in the fulfillment of their marital or familial duty, severance of social bonds and apprehension in case the person with dementia becomes angry, resentful or distressed from respite are common themes identified (45, 46). When family members, including the person receiving the care, disapprove of the respite (32, 47), the caregiver will be unlikely to take a break from their care duties. To avoid conflict, caregivers often prioritize the wishes of their relative facing the disease (48). In some cultures, the role of caregiver is seen as one of sacrifice and duty, which is a barrier to the use of respite (49) and therefore sending parents to nursing homes could be considered unfilial (50).

### Primary care physicians’ power to admit patients with dementia to intermediate care

In our survey it seems that in many countries primary Care physicians are not entitled to admit patients with dementia to a form of Intermediate care on their own and that they do need an approval from a secondary care specialist.

### Quality of intermediate care

According to our survey GPs in Switzerland are those more satisfied of the quality of intermediate care for patients with dementia. GPs also stated that structured care approach could positively impact carer burden (44). Lack of coordination among community services is another important issue (44).

### Education, training and updating of general practictioners

According to our survey many general practictioners (GPs) complain of not being informed and regularly updated and trained to the management of the intermediate care services available in their areas.

Poor uptake of social support services by carers of people with dementia is often due to a lack of awareness of their existence (51, 52). Therefore, existing dementia support services are often not efficiently linked with different stakeholders in the home-care setting (53). This situation applies to many countries worldwide (54). As a result, patients with dementia and their family caregivers are often not well informed about the services available in their community and therefore do not use the support structures efficiently (53).

GPs find providing post-diagnosis information on services and supports particularly challenging and similarly people with dementia and their family caregivers feel they need more support from their GPs in this post-diagnosis period (51). An analysis of GPs’ educational needs in a study conducted in Ireland (55) showed that GPs wanted access to up-to-date, clinical information that would help them to manage a patient with dementia and offer optimal post diagnosis care to people dementia and their families. GPs were aware of the importance of social supports, but they were often “unaware of how to access them” (55).

A Flemish study showed that GPs are often unfamiliar not only with the available detection and diagnostic possibilities, but also in post diagnostic care (56).

### Resourcing of primary care

One of the major obstacles to comprehensive care for patients with dementia in primary care is the resourcing of primary care (44).

## Conclusion

Intermediate care in Europe is diverse and heterogeneous. There is no universal access to care. Primary care physicians are not informed and are not regularly updated and trained to manage the intermediate care services available in their area. One of the biggest barriers to comprehensive care for dementia patients in primary care is primary care resourcing. In many countries, primary care physicians are not authorized to refer dementia patients to some form of intermediate care themselves but require authorization from a secondary care specialist. The main advantages are better medical care for patients, a better quality of life for caregivers, and prevention of burnout among caregivers. The major disadvantages are the high cost to the family and disorientation, and exacerbation of behavioral and psychological symptoms. Measures are needed to support the families of people with dementia, as they bear most of the costs associated with dementia care.

### Strengths and limitations

We carried out a small-scale mixed methods study and there could be limitations related to the transferability of our findings. Nonetheless, regarding the aim of addressing healthcare professionals’ perceptions about intermediate care in their daily clinical practice this objective was achieved. Since we used a convenience sample of informants the representativeness of primary care personnel for each country may be questionable although we tried to achieve geographical variation. The national coordinators tried to avoid bias and recruit practicing primary care doctors with different interests, and not necessarily in dementia or intermediate care.

Because this is a survey of key informants, we cannot fully assess the representativeness of the sample. However, to get the most accurate picture of selection bias, all researchers keep a detailed log of selection and recruitment strategies in their country. The sample is also compared as closely as possible with the national population of GP practices. Our questionnaire was refined after a first pilot study. Yet, it was not validated against other measures apart from a face validation procedure. We cannot rule out the possibility of confounding or alternative explanations to our results, since the survey responses show points of view and not actual data. We should also emphasize that differences in the number of answers to each of the questions, the online questionnaire and the selection process may be a source of independent biases in generalizability of the results.

## Data availability statement

The raw data supporting the conclusions of this article will be made available by the authors, without undue reservation.

## Ethics statement

This study was approved by (1) the Bioethics Committee in UK (Approved by the Health Research Ethics Committee of Keele University School of Medicine ref. MH-200113) and (2) Ireland (Approved by the Social Research Ethics Committee of University College Cork Log 2020-143). The patients/participants provided their written informed consent to participate in this study.

## Author contributions

CO performed statistical analysis and drafted the first version of the manuscript. CD-D, FP, AJ, and JD conceived the idea of the project. FP contributed to the project coordination and drafting a revision of the manuscript. CO and TF contributed to drafting and revision of the manuscript. CD-D, CO, TF, PT, VL, SK, LA, AK, CM, CH, GD, DK, RV, SG, VT, RF, and AM involved into the development of the final release of the questionnaire, provided the translation of the questionnaire into the national languages, recruited the respondents, and provided the back translation of the free text parts into English. All authors reviewed and approved the manuscript prior to submission.
