# Supplementary material for: Intermediate care in caring for dementia, the point of view of general practitioners: A key informant survey across Europe
Source: Front Med (Lausanne). 2022 Oct 19;9:1016462. doi: 10.3389/fmed.2022.1016462 (PMC9627031; doi:10.3389/fmed.2022.1016462)
Supplement: Supplementary file 1 [file Data_Sheet_1.pdf]

Intermediate care

Dear colleague,
we kindly ask you to fill in the following questionnaire concerning "Intermediate care in caring for people living with dementia"

Intermediate care is often defined as healthcare occurring somewhere between traditional primary (community) and secondary (hospital) care settings. Intermediate care can vary from in-home services to densely staffed nursing homes. High quality intermediate care is important in the care of complex neurocognitive disorders (dementia) and may prevent caregiver burn out. We found great variation in the use of intermediate care for persons with neurocognitive disorders across Europe in a recent European General Practice Research Network (EGPRN) survey. This study aims at investigating intermediate care services for people with neurocognitive disorders across Europe.
Kind Regards,

Ferdinando Petrazzuoli MD, PhD, EGPRN Educational Committee, Center for Primary Health Care Research, Department of Clinical Sciences, Lund University, Malmö, Sweden.

Sven Streit, GP, MD, PhD, Institute of Primary Health Care (BIHAM), Faculty of Medicine, University of Bern

Steering Committee:

Clarisse Dibao-Dina, MD, PhD, Department of General Practice, University of Tours. Project lead

Jan De Lepeleire, GP, MD, PhD ACHG University of Leuven, Belgium.

In case of any questions – please contact: ferdinando.petrazzuoli@gmail.com

*Required

1. Are you working as a General Practitioner?

   Mark only one oval.

   ( ) Yes
   ( ) No

2. If you are not working as a general practitioner you cannot complete the survey

   ____

Different types of intermediate care

1) Integrated at-home services.
The patient is cared for at home just for a few hours by social workers people from the voluntary sector et cetera, and visited at home by doctors, nurses. The familial/informal caregiver is supported in terms of clinical tasks to do but still has a 24 hours a day commitment.

2) Respite and relief services for caregivers
Respite care is short-term relief for the caregiver. The patient is cared for at home for one or two weeks by social workers people from the voluntary sector paid carer et cetera, and visited at home by doctors, nurses. The familial/informal caregiver is supported not only in terms of clinical tasks to do but he/she is given also free time to get out of the house because the task of caregiver is taken over temporarily by someone else.

3) Day care centres
The patient is taken to a different place but just for a limited period of time during the day. During this time the familial/informal caregiver is free to do what he/she wants.

4) Nursing homes and Residential Home
(In the nursing home the staff is the staff is present 24/7. In the residential home the staff is involved only for a limited period of time a day)
The patient is staying at a different location all the time. No need for familial/informal caregiver.

Skip to question 3

Advantages and disadvantages of intermediate care

3. 1)What do you think could be the main advantages of well administered intermediate care in a patient with neurocognitive disorder (dementia)? *

Tick all that apply.

- [ ] Better treatment and care of the patient
- [ ] Better quality of life for caregiver
- [ ] Prevention of caregiver burnout
- [ ] A break and relief for the caregivers
- [ ] Patient choice
- [ ] Others

4. Please describe other advantages

____
____
____
____
____

5. 2)What do you think are the main DISADVANTAGES of intermediate care in a patient with neurocognitive disorder (dementia)? *

Tick all that apply.

- [ ] Poorer treatment and care of the patient
- [ ] Poorer quality of life of caregiver
- [ ] Poorer prognoses of the patient
- [ ] Disorientation and exacerbation of behavioral and psychological symptoms of dementia (BPSD) because the patient has been moved into another setting
- [ ] Sense of shame of family caregivers (not being able to care on their own with natural obligation according to social conventions)
- [ ] High cost for the family
- [ ] High cost for the Health Care System and Social Care System
- [ ] Other

6. Please describe other disadvangates

Availability

7. 3)What kind of intermediate care is available in your country? *

Tick all that apply.

- [ ] Integrated at-home services
- [ ] Respite and relief services
- [ ] Day care centres
- [ ] Nursing homes and Residential Home
- [ ] Other

8. Describe other options

9. 4)Are intermediate care services homogeneously available in your country? /(homogeneously means: no difference between regions and no difference between rural and urban setting) *

Mark only one oval.

	1	2	3	4	5	
Not at all	◯	◯	◯	◯	◯	Very much

10. Please describe if there are big regional differences or differences between rural and urban settings.

11. 5)Are these two types of intermediate care (Integrated at-home services/Respite and relief services for caregivers, described in full in question 1) provided for free in your country? (Free means that the patient is not charged or he is reimbursed by the health care system) *

Mark only one oval.

	1	2	3	
Not at all/patiens are charged and no reimbursement is expected	◯	◯	◯	Yes completely free/or completely reimbursed

12. Please describe

13. 6)Do families receive economic support when a patient is admitted to a nursing home or Long-term Care Facility (type 3 and 4 in question 1) in your country? *

Mark only one oval.

	1	2	3	
Not at all/patients are charged and no reimbursement is expected	◯	◯	◯	Yes completely free/or completely reimbursed

14. If yes, - how much economic support per in terms of percentage? (100%, 80%, 50%) . Please describe *

15. 7)Does your country have written standards or guidelines for admission to any form of intermediate care? *

Mark only one oval.

- Yes
- No
- other

16. Describe

17. 8)What is in your opinion the general attitude of family caregivers towards admitting their relative to a nursing home in your country? *

Mark only one oval.

	1	2	3	4	5	6	7	8	9	10	
Happy											Ashamed

18. Describe

19. 9)Do you as a GP have the power to admit a patient to any form of institutional support of intermediate care? *

Mark only one oval.

- Yes
- No
- Other

20. Please describe which ones

21. 10)Do you, as a GP, need an approval from a secondary care specialist or any other authority to admit a patient to any form of institutional support? *

Mark only one oval.

- Yes
- No
- Other

22. Describe

23. 11)How do you rate the quality of intermediate care for persons living with dementia in your country?

Mark only one oval.

	1	2	3	4	5	
Low quality						High quality

24. 12)What are in your opinion the main issues related to intermediate care for persons living with dementia in your country or in your area? (Tick all that apply) *

Tick all that apply.

- Limited financial support
- Low quality of the service
- Difficult to access with difficult bureaucratic procedures
- Stigma for the family (for the nursing home)
- Feeling that the situation is getting worse in the last few years, because of cuts in health care expenditure
- Other

25. Describe

26. 13)In your area are GPs educated and trained to get acquainted with and manage intermediate care services? *

Mark only one oval.

	1	2	3	
Not at all	⬭	⬭	⬭	Highly educated and trained

27. Please describe how

28. 14)In your area are GPs regularly updated on the intermediate care services available? *

Mark only one oval.

	1	2	3	
Not at all	⬭	⬭	⬭	Regularly updated

29. Please describe how

Respondent details

30. 15) Your gender *

Mark only one oval.

⬭ Male

⬭ Female

31. 15) Your age *

32. 17) Country *

33. 18) Where do you practice family medicine? (You can choose only one answer)

Mark only one oval.

- Urban
- Semirural
- Rural

34. 19) How many years have you been practicing as a general practitioner? (Please insert a number)

____

35. 20) Would you be interested in another survey on Rented Caregiver also defined as "directly employed care workers" (who are usually foreign domestic helpers)?

Mark only one oval.

- yes
- no

This content is neither created nor endorsed by Google.

Google Forms
